# Capability, opportunity and motivation for shared decision‐making about valproate as an antiseizure medication treatment for epilepsy in women with pregnancy potential: A qualitative study of patient perspectives

**DOI:** 10.1111/bjhp.70045

**Published:** 2025-12-19

**Authors:** Sarah Louise Griffiths, Delyth James, Denitza Williams, Lynette James, Andrew Evans, William O. Pickrell, Christine McKnight, Sarah Brown, Rhiannon Phillips

**Affiliations:** ^1^ Cardiff School of Sport and Health Sciences Cardiff Metropolitan University Cardiff UK; ^2^ Department of Pharmacology and Therapeutics, Institute of Systems, Molecular, and Integrative Biology University of Liverpool Liverpool UK; ^3^ School of Medicine Swansea University Swansea UK; ^4^ Division of Population Medicine, School of Medicine Cardiff University Cardiff UK; ^5^ Cardiff and Vale University Health Board Cardiff UK; ^6^ Health and Social Services Group Welsh Government Cardiff UK; ^7^ Swansea Bay University Health Board Swansea UK

**Keywords:** Capability Opportunity Motivation Model of Behaviour (COM‐B), epilepsy, preconception planning, reproductive health, shared decision‐making (SDM), sodium valproate

## Abstract

**Objectives:**

Valproate is a highly effective antiseizure medication but carries significant teratogenic and neurodevelopmental risks to offspring if used during pregnancy. A shared decision‐making (SDM) approach is recommended to guide clinician/patient discussions on valproate suitability for women with pregnancy potential. This study applied the Capability, Opportunity, Motivation–Behaviour (COM‐B) theoretical framework to explore barriers and facilitators to SDM in valproate prescribing from the perspectives of women with epilepsy who have pregnancy potential.

**Design:**

Qualitative study using timeline‐facilitated semi‐structured interviews informed by the COM‐B model.

**Method:**

Twelve UK‐based women 18–50 years (M_age_ = 33.3, SD = 7.59) prescribed valproate were recruited via pharmacies and epilepsy organizations' social media. Interviews were thematically analysed and interpreted using the COM‐B model.

**Results:**

Participants were highly motivated to engage in SDM behaviour but reported limited opportunities. Challenges to COM‐B domains included insufficient information exchange, low confidence navigating complex epilepsy/reproductive health care discussions, and tensions navigating valproate risks and benefits within broader contexts of seizure control and reproductive health. Initial prescribing during acute seizure crises may have precluded meaningful collaborative discussion. Valproate prescribing/deprescribing incongruent to reproductive goals often resulted in deep regret and deleterious health outcomes for women (and children exposed to valproate in utero).

**Conclusion:**

Comprehensive SDM when valproate is considered clinically appropriate could support informed, patient‐centred decision‐making. Equipping clinicians to navigate multifaceted risk/benefit discussions and empowering patients with clear, tailored information can help ensure treatment decisions align with reproductive goals. This study highlights the need to embed SDM in valproate prescribing consultations and throughout treatment duration.

**Statement of Contribution**

**What is already known?**

- Valproate is a highly effective antiseizure medication but poses significant foetal risks in pregnancy.
- Shared decision‐making (SDM) supports patient‐centred treatment that aligns with reproductive goals.
- Women with epilepsy often experience a lack of consistent reproductive health support.

**What this study adds?**

- Identifies key barriers and facilitators to SDM in valproate prescribing through the COM‐B lens.
- Highlights limited opportunity and capability to engage in collaborative SDM valproate discussions.
- Shows motivation to engage in SDM is strong but is undermined by limited opportunity and support.

## INTRODUCTION

Epilepsy is a common chronic neurological condition characterized by recurrent, unprovoked seizures resulting from excessive, hypersynchronous and irregular brain activity (Beghi, 2020; Epilepsy Society, 2024; Fisher et al., 2014). Epilepsy affects an estimated 1% of the global population (approximately 70 million individuals worldwide) and contributes to 0.5% of the global burden of disease (Thijs et al., 2019; World Health Organization, 2024). The primary treatment for epilepsy is antiseizure medication (National Institute of Clinical Excellence, 2022, updated 2025). Around a third of people with epilepsy have idiopathic (genetic) generalized or unclassifiable epilepsies, for which effective treatment is limited (Marson et al., 2007, 2021).

Sodium valproate (valproate) is a broad‐spectrum, highly effective antiseizure medication with superior efficacy in treating idiopathic (genetic) generalized epilepsies (Marson et al., 2007, 2021; Patsalos & St Louis, 2018). In addition to epilepsy, valproate is licensed for the treatment of bipolar disorder and is used off‐label (outside of its product licence) for migraine prophylaxis, neuropathic pain, dementia and depression (National Institute for Health and Care Excellence [NICE], 2025a, 2025b).

Valproate is among the most teratogenic agents used in neuropsychiatric pharmacotherapy, carrying a significantly higher risk of teratogenicity compared to other antiseizure medications (Andrade, 2018; Bromley, 2016; Tomson et al., 2011, 2015). Prenatal exposure to valproate presents a risk of congenital malformations in up to 1 in 9 infants and neurodevelopmental disorders in as many as 4 in 10 (Medicines and Healthcare products Regulatory Agency [MHRA], 2023a, 2023b, 2024).

Additionally, a retrospective observational post‐authorization safety study (PASS, Bierrenbach, 2020) suggested a possible association between paternal valproate exposure during spermatogenesis (i.e. within 3 months prior to conception) and an increased risk of neurodevelopmental disorders in offspring, compared with fathers prescribed lamotrigine or levetiracetam. In response, UK and European medicine regulators advise that men taking valproate should be informed of the possible risk of neurodevelopmental disorders and are recommended to use effective contraception during valproate treatment and for at least 3 months after discontinuing valproate (European Medicines Agency, 2023, 2025; MHRA, 2023a, 2023b, 2024). Furthermore, UK guidelines recommend men are informed of the possible risk of valproate use to male fertility (MHRA, 2024). However, recent large‐scale retrospective population studies have shown no significant differences in male fertility parameters associated with valproate use in men with epilepsy or bipolar disorder (Mbizvo et al., 2025), and no increased risk of congenital malformations or neurodevelopmental disorders in offspring of men with epilepsy who were prescribed valproate during spermatogenesis (Christensen et al., 2024; Honybun et al., 2025). However, the present study focuses only on experiences of valproate prescribing as a treatment for epilepsy for women with pregnancy potential.

Since 2018, UK policy has progressively tightened restrictions and guidance on valproate prescribing for women of childbearing potential (Gaudio et al., 2022; MHRA, 2018, 2024, 2025); currently in the UK, valproate must not be initiated in patients (male or female) under 55 years unless two specialists independently confirm that there are no other effective or tolerated alternative treatments (MHRA, 2024, 2025). All post‐menarche and premenopausal women prescribed valproate must meet the conditions of the Pregnancy Prevention Programme (PPP), including the uninterrupted use of highly effective contraception (<1% failure rate [MHRA, 2019]) for the duration of treatment, unless the prescribing clinician determines there are compelling reasons to conclude there is no risk of pregnancy (MHRA, 2024). Patients must also attend an annual risk review assessment and sign an Annual Risk Acknowledgement Form to confirm they are aware of the risks and the requirement to adhere to the PPP if valproate is continued (MHRA, 2024).

Critically, there may be circumstances where no alternative antiseizure medications are effective or tolerated by women with pregnancy potential and where valproate remains the only clinically appropriate option to achieve seizure control, offering the best quality of life (Angus‐Leppan et al., 2024; Patsalos & St Louis, 2018; Shakespeare & Sisodiya, 2020; Vajda et al., 2020; Watkins, Cock, Angus‐Leppan, Morley, et al., 2019; Watkins, Cock, Angus‐Leppan, & Shankar, 2019).

Valproate withdrawal in patients with well‐controlled epilepsy is linked to an increased risk of breakthrough seizures, emergency department visits, hospital admissions, injuries, falls and new‐onset depression (Mbizvo et al., 2024). Between 33% and 43% of clinicians reported a deterioration in seizure control among women who switched from valproate to alternative antiseizure medications (Angus‐Leppan et al., 2020). Therefore, it is essential that women are informed of the risks of valproate withdrawal/switching when providing consent to change treatment, underscoring the challenges of balancing valproate's benefits (i.e. seizure control) with its potential risks (Angus‐Leppan et al., 2020, 2024; Angus‐Leppan & Liu, 2018; Kirkpatrick et al., 2021; Macfarlane & Greenhalgh, 2018; Winterbottom, 2012). Given the complexity of decisions surrounding valproate prescribing, a shared decision‐making (SDM) approach has been advocated (Macfarlane & Greenhalgh, 2018; NHS, 2024; Pickrell et al., 2015; Turner et al., 2019; Winterbottom, 2012).

SDM is an interactive, collaborative process in which patients and health care professionals (HCPs) jointly make health care decisions, partnering clinical expertise with the patient's intrinsically individual values, perspectives and preferences (Elwyn et al., 2012). SDM models typically involve describing treatment options (creating choice awareness), eliciting patient preferences, tailoring information, supporting deliberation and incorporating patient input into the final plan (Bomhof‐Roordink et al., 2019; Elwyn et al., 2012). However, the behavioural factors that influence patient participation in valproate‐related SDM remain poorly understood.

To underpin the development of SDM interventions, it is essential to understand what decisional support patients need, identify barriers and facilitators to engagement in SDM, and explore how communication between HCPs and patients shapes decision‐making (NICE, 2021a). Previous qualitative research indicates that women with epilepsy often receive inadequate or inconsistent reproductive health counselling and lack support on topics such as highly effective contraception, pregnancy planning and the teratogenic effects of some antiseizure medications (Kirkpatrick et al., 2022). A joint 2022 survey by Epilepsy Action, Epilepsy Society, and Young Epilepsy found that 9% of women prescribed valproate were unaware of the risks of birth defects and neurodevelopmental disorders in children exposed to valproate in utero (Epilepsy Society, 2023).

Women with epilepsy often express a preference for HCPs to initiate discussions about reproductive health, ideally beginning in adolescence, with an emphasis on clear, supportive communication and evidence‐based decisional support regarding the risks and benefits of antiseizure medications (Kirkpatrick et al., 2021; Manski & Dennis, 2014). Women also report significant psychological distress (e.g. worry and fear) related to epilepsy and pregnancy planning, including concerns about teratogenic antiseizure medication, pregnancy outcomes (including seizure control during pregnancy), and future child health (Winterbottom, 2012), often exacerbated by uncertainty, perceived personal responsibility, and inconsistent reproductive healthcare support (Lawther et al., 2018). It is widely recognized that women with epilepsy should have access to preconception counselling to reduce both maternal and foetal risks when pregnancy is a reproductive goal, thus supporting risk reduction, informed decision‐making and engagement in SDM (Pegg et al., 2024; Winterbottom, 2012).

The Capability, Opportunity, Motivation model of Behaviour (COM‐B) provides a clinically informed, structured theoretical framework for systematically understanding the determinants of health behaviours (Michie et al., 2011). Capability refers to the psychological and physical ability to perform a behaviour, opportunity refers to social and physical/environmental factors, and motivation refers to conscious and unconscious processes that drive behaviour (Michie et al., 2011). By observing or analysing these three components, the barriers and facilitators to specific behaviours (e.g. SDM) can be identified (Michie et al., 2011). The COM‐B model has been used to understand participation in SDM in other chronic conditions that may impact reproductive health in women, including cystic fibrosis (Williams et al., 2023) and chronic kidney disease (McLaughlin et al., 2023).

Women prescribed valproate often face complex reproductive healthcare dilemmas, including decisions about highly effective contraception, preconception and pregnancy planning, seizure management and conflicting priorities (e.g. seizure control priorities versus desire for pregnancy), and pressure from self or others regarding reproductive choices (e.g. to avoid or achieve pregnancy; Lawther et al., 2018). It is equally critical to acknowledge that uncontrolled seizures carry significant risks of morbidity and mortality, including sudden unexpected death in epilepsy (SUDEP), and that previous work has highlighted concerning deficits in the information provided to patients regarding seizure‐related risks of valproate withdrawal or withholding (Hanna et al., 2024).

When valproate is a clinically appropriate treatment option, SDM can support informed, value‐aligned choices by fully considering a woman's reproductive goals and should be integrated throughout the reproductive years (Robson et al., 2020). However, despite its recognized benefits, SDM is inconsistently implemented (Department of Health and Social Care, 2021b). Systemic pressures, such as financial targets and productivity, may hinder collaborative patient/HCP decision‐making and (ultimately) patient safety (Hughes, 2024). Women with epilepsy continue to report that they are not receiving adequate information about the risks of valproate, putting potential future offspring at risk (Epilepsy Society, 2023; Swanborough, 2020).

This study aimed to explore the barriers and facilitators to participating in SDM from the perspectives of women with epilepsy who had pregnancy potential, for whom valproate had been a clinically appropriate treatment option, using the COM‐B model as a theoretical framework.

## METHOD

### Study design

A qualitative study design was adopted using timeline‐facilitated one‐to‐one semi‐structured interviews (Bagnoli, 2009; Pell et al., 2020). Timelines are a visual research method used alongside interviews to help participants reflect on and organize important life events chronologically (Berends, 2011). Timelines illustrate not only the sequence of experiences but also the meanings participants assign to them (Berends, 2011). This approach supports the recall and organization of personal histories and can help situate clinical issues within broader life contexts (Gramling & Carr, 2004). Additionally, given the social stigma associated with epilepsy and the potential marginalization of people living with epilepsy (Stephen et al., 2019), visual timelines were used to support participant empowerment and reduce any perceived interviewer/interviewee power imbalances during interviews (Goldenberg et al., 2016). The study used theory‐informed thematic analysis, guided by the COM‐B model as the methodological orientation.

### Participants and sampling

Participants were purposefully recruited through a multipronged approach of convenience and snowball sampling. Women with epilepsy, aged 18–50 years, who had been prescribed valproate or who had discussed it as a treatment option for epilepsy were recruited via adverts distributed in Welsh community pharmacies and UK‐based epilepsy support organizations' social media. The age range of interest aligns with previous research on patient benefit/risk understanding of antiseizure medications in women of reproductive potential (e.g., Holmes et al., 2019). Participants were not known to the research team before recruitment.

Community Pharmacy Wales (the body responsible for representing all pharmacies in Wales on NHS matters) provided the research team with a list of community pharmacies in Wales registered with the National Electronic Claim and Audit Form (NECAF) system as dispensing valproate to women and girls aged between 13 and 50 years between April and August 2022. Twelve pharmacies across five of the seven Health Boards in Wales agreed to advertise the study. Pharmacies displayed recruitment posters on‐site and were provided with printed flyers to distribute to patients receiving epilepsy medications, including but not limited to valproate. Additionally, UK epilepsy support organizations (Epilepsy Action, Epilepsy Connections, Young Epilepsy, and the Organization for Anti‐Convulsant Syndrome) agreed to advertise the study via social media (e.g., Twitter™, Facebook™, Instagram™). All adverts for the study included a URL and QR code linking to a Qualtrics™ eligibility screening survey.

Inclusion criteria: epilepsy diagnosis; assigned female at birth; current or previous use of valproate or prior discussion of its suitability with a HCP; aged 18–50 years; able to complete an interview in English or Welsh; UK resident. Exclusion: inability to provide informed consent. Inclusion criteria were participant‐reported (no medical records were reviewed). The research aimed to develop an in‐depth understanding of the views and experiences of women for whom valproate had previously been a clinically appropriate antiseizure medication. The study sought representation from a clearly defined but relatively small population, and those facing decision‐making around valproate often navigate complex, deeply personal decisions with significant implications for their own health and well‐being, and in some cases, for future pregnancy or offspring. Consequently, the study was guided by the concept of *information power* (Malterud et al., 2016) rather than the traditional notion of data saturation. The study's purpose was not to produce generalizable findings, as expected with quantitative research, but rather to generate rich and nuanced insights into a complex, underexplored area of clinical decision‐making. According to the framework of information power, when a study has a narrow aim, includes a specific sample, is theoretically informed and involves high‐quality dialogue, a smaller number of participants can still provide sufficient depth and relevance of information to address the research question. The greater the relevance of the sample's contribution to the study objectives, the fewer participants required (Malterud et al., 2016). This approach was chosen as it prioritizes the richness and relevance of the data in relation to the study's aims, rather than seeking data redundancy, making it well‐suited to theoretically informed and in‐depth qualitative inquiry (Malterud et al., 2016).

A total of 12 participants were recruited, providing a range of ages, reproductive goals and treatment experiences, consistent with the aim of achieving sufficient information power (Malterud et al., 2016). All participants gave their informed consent to take part in the study. Participant details can be found in the results section.

### Materials

A semi‐structured interview schedule was developed to explore participant views/experiences of valproate use, accuracy of information received/recalled regarding risk of valproate use in pregnancy at initial prescription and during routine/annual reviews, decisional support regarding reproductive preferences, and participant attitudes towards valproate‐SDM. The interview schedule was developed through an iterative process and was not based on the review of the literature (as, to the best of the authors' knowledge, no study had utilized the COM‐B framework to explore this area previously). Instead, the interview schedule was developed with consideration of the COM‐B theoretical framework (i.e. designed to explore capability, opportunity and motivation to engage in valproate‐focused SDM). The interview schedule was piloted with a pseudo‐patient (a health psychology master's student), with revisions informed by the multidisciplinary research team to ensure clarity, relevance, and alignment with the study's aims.

Ahead of interviews, participants were sent a timeline template along with a (fictitious) example to support completion (a copy of a blank timeline and the example is available to view in File S2). During interviews, participants were shown a Welsh Government educational video on the risks of foetal valproate exposure in‐utero to elicit their views on the integration of such media within the patient care pathway (All Wales Therapeutics and Toxicology Centre, 2023).

### Procedure

Interviews were conducted online between January and August 2023 via Microsoft Teams™. One researcher (SG, female Senior Research Assistant from the Cardiff School of Sport and Health Science, Cardiff Metropolitan University, BSc Psychology, MSc Health Psychology) conducted all interviews privately from a secure, non‐clinical location. Participants joined from a location of their choosing. Only the interviewer and interviewee were present. SG had post‐graduate training and experience conducting qualitative interviews on sensitive health‐related topics. Participants were made aware of her research background and interest in SDM and antiseizure medication prescribing to women with epilepsy of pregnancy potential. Participants were unknown to the research team and were introduced to SG via email during the recruitment stage.

Participants were encouraged before interviews that they did not have to discuss anything they were uncomfortable with and could complete the timeline with as much or as little detail as they wished (or omit completely if preferred). The timeline was incorporated to foster participant agency, prompt reflection, and support discussion (McLaughlin et al., 2023; Pell et al., 2020; Williams et al., 2023), while the semi‐structured interview schedule guided the interviews.

Exploring the participant‐completed timeline enabled the interviewer to affirm the participant's agency over their narrative and use it to steer the interview (Goldenberg et al., 2016; Pell et al., 2020). Interviews explored participants' reproductive healthcare experiences, evolving goals across life stages, and interactions with HCPs regarding valproate prescribing and reproductive planning. Discussions also examined information exchange and service provision related to reproductive health management, including preconception, pregnancy, and inter‐pregnancy planning. Following discussions about their initial valproate prescriptions and earlier consultations, participants viewed a Welsh Government educational video about valproate (All Wales Therapeutics and Toxicology Centre, 2023) to explore the media's potential role in supporting SDM. Participants were then invited to share their impressions, reflect on whether they had received similar details of information at the time of initial valproate prescription, and consider how such media could be integrated into valproate‐related information provision/consultations.

All interviews were video‐recorded and transcribed verbatim. Participants received a £20 shopping voucher as a token of appreciation. Participants were able to withdraw consent up to 7 days post‐interview, after which data were anonymised to ensure confidentiality. Only one participant completed a follow‐up interview to discuss a subsequent annual valproate risk review. Transcripts were not returned to participants to avoid post‐interview bias and burden (Hagens et al., 2009).

### Analysis

Field notes and participant‐generated timelines were reviewed multiple times, with written reflections completed shortly after each interview to ensure contextual accuracy. While these materials were used to support contextual understanding, they were not subjected to formal analysis. Interview recordings were transcribed verbatim, anonymised, and pseudonyms were allocated. Data were coded by a single researcher (SG) using NVivo™ (version 12). Thematic analysis was conducted to interpret patterns of meaning within the interview recordings and transcripts (Braun & Clarke, 2006). A six‐step approach of data familiarization, generating initial codes, searching for themes, reviewing themes, defining and naming themes and reporting findings was followed (Clarke et al., 2015).

A reflexive and iterative analytical approach was employed, with ongoing comparison of emerging patterns against the original data to ensure thematic coherence and depth, thereby enhancing the rigour of the findings (Saunders et al., 2018). Initial coding, guided by the study objectives and research questions, used a hybrid deductive‐inductive approach (Braun & Clarke, 2022), facilitating the identification of both theory‐driven and participant‐driven themes. This approach enabled the development of a hierarchical coding structure, integrating data‐driven and theory‐based interpretations. The coding tree consisted of overarching parent themes, each encompassing several subthemes that captured nuanced aspects of participant experiences. The COM‐B theory was used as an interpretive lens to understand the facilitators and barriers in participant lived experiences of SDM at the intersection of valproate prescribing and reproductive healthcare; themes identified during analysis were mapped against the COM‐B domains (capability, opportunity, and motivation). Collaborative discussions (a triangulation method) with the multidisciplinary research team facilitated the refining of themes identified and enabled primary and secondary categorization (Clarke & Braun, 2013). Collaborative discussion enabled the evaluation of identified themes (Barbour, 2001; Liamputtong & Ezzy, 2005; Robson, 2002).

### Reflexivity

Please see File S3 for details of reflexivity.

### Reporting standards

This study was reported in accordance with the Consolidated Criteria for Reporting Qualitative Studies (COREQ) checklist (Tong et al., 2007; File S4). The Contributor Role Taxonomy (CRediT) was also completed for this manuscript (National Information Standards Organization, 2022; File S5).

## RESULTS

A total of 53 individuals responded to the recruitment call and undertook an initial Qualtrics™ screening survey; 18 respondents met all eligibility criteria, 15 responded to further communication, and 12 were interviewed. Ages ranged from 23 to 46 years (M = 33.3, SD = 7.59). All participants self‐identified as women and as being of White Welsh/English/Scottish/Northern Irish/British, or White Irish ethnicity. Interviews lasted between 42 and 93 min (M = 62.2, SD = 14.74).

**FIGURE 1:**
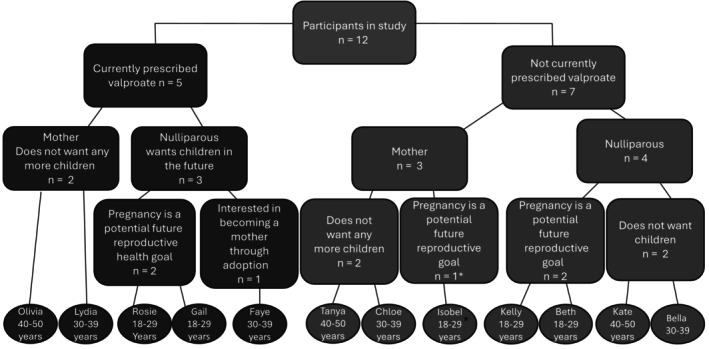
Participant valproate status, family planning status, and future reproductive health goals. *At time of interview, participant was waiting for neurology referral for preconception consultation/counselling before embarking on her next pregnancy.

All 12 participants had previously been prescribed valproate; five were currently prescribed valproate. Five participants were mothers through childbirth, four of whom did not wish to have more children. The remaining seven participants were nulliparous; of these, five expressed a desire to have children in the future, one of whom expressed an interest in becoming a mother through adoption, while two wished to remain child‐free by choice. Three nulliparous women who hoped to become mothers through pregnancy in the future were currently prescribed valproate (see Figure 1 [pseudonyms used throughout]).

**FIGURE 2:**
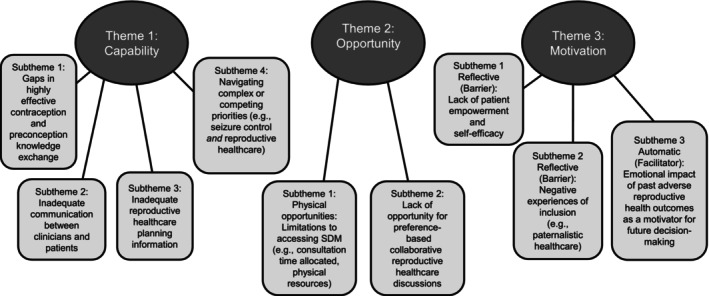
Thematic analysis findings mapped onto the COM‐B framework. SDM, shared decision‐making.

Three superordinate themes—capability, opportunity and motivation—were identified in relation to accessing SDM for valproate use within the context of reproductive healthcare. These themes encompassed nine subthemes, each reflecting identified barriers or facilitators to collaborative discussions about treatment suitability (see Figure 2). All themes and subthemes were mapped onto domains of the COM‐B model to support interpretation.

### Theme 1: Capability

The capability theme relates to participants' perceived experiences of internal psychological and external physical factors that influenced their ability to engage in SDM during valproate‐focused consultations. This included how HCPs communicated information to them, their access to relevant information, and their confidence in navigating complex healthcare consultations. Four subthemes were identified.

#### Subtheme 1: Gaps in highly effective contraception and preconception knowledge exchange

Although some participants recalled discussing the risks of valproate use in pregnancy with their HCP and the importance of pregnancy prevention, others felt they had not received enough information to make a fully informed decision. Several also reported limited guidance on optimal methods of highly effective contraception.

I wasn't informed about anything…but I [now] know, the pill… [isn't highly effective contraception] … so, you need a secondary one [barrier contraception] …if you don't know the pill isn't 100%…who's gonna tell you? (Tanya)

Some participants reported a lack of detailed risk information during initial valproate‐prescribing consultations, leaving them feeling [on reflection] inadequately informed about its risks in pregnancy.

They just put me on it [valproate], increased it a few times, and that was it. They didn't say anything, they didn't tell me about side effects, pregnancy, they didn't tell me about nothing. It was, take this, it works, off you pop. (Olivia)

There was a brief mention about “Ohh we just need to obviously think about the future and family planning if you were on valproate”, and that was very much it. There was no real expression of concern as such, or you know this is really important… So, I didn't realise the risks involved, I think, because they weren't really explained properly [at initial prescription]. (Isobel)

Participants described their understanding of the risks associated with valproate as something that developed over time through ongoing clinical consultations or personal research. Many reflected that receiving more comprehensive information earlier in treatment, particularly at the point of initial prescription, could have better supported informed decision‐making from the outset.

I got most of my information about sodium valproate by looking for it myself…it wasn't explained to me well. Most of the knowledge I have is from my own kind of research…the way [HCPs share initial information] could be improved massively. (Kelly)

#### Subtheme 2: Inadequate communication between HCP and patients

Poor communication about reproductive healthcare was frequently contextualized in terms of inadequate contraception advice or lack of information around the risks of valproate use in pregnancy; reproductive healthcare communication was not sufficiently clear for participants to feel they had made informed decisions. Communication had, at times, been poor, particularly around the suitability of contraception. Unclear or incomplete information led participants to retrospectively question whether their contraceptive decisions had been fully informed.

I was on the pill from…17 [to] 23, being assured by my GP this is safe for me to use…It wasn't until I was 23, having my last neurology appointment… I found out [the pill is not ‘highly effective’ contraception] … if I wasn't having protected sex, I could have easily gotten pregnant. (Beth)

After discussing their prior experiences with HCPs regarding valproate risk communication, participants were shown a Welsh Government‐produced video stating the risks of valproate use during pregnancy and explaining the premise of the PPP. The video was well received by most participants, who felt that they, along with family/carers/significant others, could have benefited from such clearly presented information during their initial valproate consultation.

I liked that it was succinct, like it was short. And I liked that it was clear and concise… like the statistics, the way the graphic shows …4 in 10, or 1 in 10…you can evaluate the risk. (Bella)

[The video could] give you more chance to think of some questions…give you a bit more time to absorb it and then think [of] some questions for when you actually go in [to the consultation]. (Rosie)

Participants noted that the language used in many written valproate patient information resources might not be accessible to people with low reading or health literacy skills, or those with additional support needs, and welcomed video‐based media as a more inclusive method of information sharing.

There's no big words in there that you sort of then have to go away and look up, you know, what does that really mean? It's all done in plain English, you understand it. (Kate)

#### Subtheme 3: Inadequate reproductive healthcare planning information

Participants' reproductive healthcare goals varied widely, including diverse motivations for contraception (including permanent sterilization), family planning, preconception planning, and consideration of adoption as an alternative to pregnancy. Participants cited multiple intersecting physiological, psychological, and social factors, highlighting the complexity of navigating reproductive healthcare alongside antiseizure medication management.

While at the time of interview all participants were aware of the importance of preconception planning, many described how their understanding had developed over time since their initial valproate prescription. Few recalled being actively supported to access relevant information or preconception planning services, with several noting they had been told to ‘plan for pregnancy’ without accompanying guidance or signposting to appropriate services

It was… if you want to get pregnant, let us know… it's very like open to interpretation… like at what stage, like how far in advance would you need to be having this conversation with your neurology team? (Bella)

Participants expressed a strong desire for clear, practical guidance on the timeframes and processes required for safe valproate withdrawal and transition to alternative antiseizure medication (if clinically appropriate) when planning for pregnancy.

I would like to know how long I have to be off it [valproate] in order to have children… when is going to be the right time to come off? … How do I come off this in a safe way?… How long do I have to be off it before it's completely out of my body? (Gail)

Concerns about the timeframe required to plan for pregnancy, along with a lack of clarity about what preconception planning involved were common among participants; the topic was recurrent and evident even among those who had experienced pregnancy(ies) exposed to valproate.

Some kind of idea as to how early on you need to start thinking about planning for families [pregnancy] and things like that would be quite informative for people that are thinking about having children… I still wouldn't have any idea today. (Isobel [who conceived three pregnancies when prescribed valproate and the contraceptive pill])

Some individuals felt disempowered and uncertain about their ability to achieve their reproductive goals and were overwhelmed by feelings of fear over the long‐term impact of valproate on their reproductive health plans.

I'm thinking things like, OK, well, what, what's the point of having my own children…but then I think, well, other people do… why am I being robbed of this? (Gail)

Some individuals recalled repeatedly requesting the discontinuation of valproate (and transition to alternative antiseizure medication) in preparation for future, sometimes distant, pregnancy, but were repeatedly advised to continue with valproate due to HCPs concerns about maintaining seizure control.

I've already had those conversations with neurologists where they didn't look at putting me on something different yet…It's a worry for me, I wanna be coming off it [valproate]… it was never the neurologist's primary concern; they would kind of very much brush it aside and that, that would be kind of the end of it. It would be kind of like, well you're doing university now so we're not gonna take you off it now. (Kelly)

However, others expressed aggrievement when valproate treatment was refused in favour of alternative antiseizure medications, particularly when valproate use was not in conflict with reproductive goals.

I thought it [valproate] was actually controlling my seizures better than just the Tegretol [carbamazepine]… I never wanted children, but it was a thing of him literally saying to me, ‘Yeah, but you might get pregnant and so you can't take it anymore.’ And that was sort of the end of his discussion with me about it. (Kate)

#### Subtheme 4: Navigating complex or competing priorities (e.g. seizure control and reproductive healthcare)

Valproate was frequently introduced following acute events, such as first or significant seizures, when participants felt uncertain, distressed, and overwhelmed with epilepsy‐related information. Participants reflected that reproductive goals were typically ‘not on their radar’, as seizure control and managing their epilepsy took priority.

I was getting asked by doctors if I'm planning to get pregnant…not only are you telling me, hey, you've got this lifelong condition, mentioning about SUDEP and you might die during your sleep…I had to think about the future straight away…I didn't fully understand what was going on…I've just known I was sick. (Beth)

Some participants reflected that warnings about the risk of valproate use in pregnancy might have been missed during life‐threatening health crises. Contemporaneously, others reported that they felt their concerns about seizure control were secondary to HCPs' concerns about pregnancy prevention. Several described spending valuable consultation time defending their ability to prevent pregnancy in order to secure valproate prescriptions. Some felt that annual risk review procedures and pregnancy‐related discussions detracted from attention to their ‘actual health’ (i.e. epilepsy‐related) concerns. Participants often viewed reproductive health and seizure control as distinct, largely unrelated and somewhat disconnected areas of health.

It sometimes feels like all the pregnancy prevention stuff distracts from…talking about my epilepsy…it eats into the consultation time… it sometimes feels like that takes precedent over my health. (Faye).

However, participants frequently reflected that had they better understood the risks of valproate exposure in‐utero they would have better understood why HCPs focused so repeatedly on pregnancy prevention.

Like, God, it was only a couple of years ago… I looked it up and then read about it…sodium valproate, and I'm like, wait…what do you mean…makes sense why he [HCP] took me off [valproate]… I didn't realise it was that bad. (Beth)

### Theme 2: Opportunity

Two subthemes were identified within opportunity.

#### Subtheme 1: Physical opportunities: limitations to accessing SDM (e.g. consultation time allocated, physical resources)

Participants identified service‐level barriers that limit opportunities to engage in SDM. Short appointment times frequently constrained comprehensive and collaborative discussion and decision‐making regarding treatment options and reproductive healthcare planning. A lack of continuity of care further inhibited collaborative decision‐making, whereas consistent contact with a familiar HCP was seen as a key facilitator, allowing clinicians to better understand and recall individual preferences and goals. Participants suggested that integrating family planning services into epilepsy clinics—such as through multidisciplinary teams—could support complex decision‐making when epilepsy specialists face time constraints. Several expressed regret over missed opportunities for meaningful discussions about preconception planning, which they felt had impaired their ability to exercise reproductive choice and attain reproductive goals.

I'm in chemical menopause now, and I've not had a separate consultation with my neurologist…I'm waiting for a hysterectomy…it's something that should have been done a long time ago… when I was at that stage in my life…when I could have had [more] children. (Chloe)

#### Subtheme 2: Lack of opportunity for preference‐based collaborative reproductive healthcare discussions

Participants frequently reported feeling that the annual valproate risk review process needed to facilitate more meaningful collaborative discussions that explored future reproductive goals to check if goals had changed. Participants reflected that they typically received little signposting on where to seek guidance between annual reviews. Many perceived the reviews as a tick‐box exercise and expressed frustration at the lack of depth and individualized or tailored discussion.

I left that [Annual Valproate Risk Review] appointment feeling so angry because he [HCP] [said] “I have to go through this Pregnancy Prevention Programme” and rolled his eyes… and said, “basically, you just need to have a quick look through these, put your initials next to the box, and then just sign at the bottom”… he still didn't actually talk through the points… over the next couple of years, I had a telephone call, and it was a very brief “is everything still the same? Yes? I've got the Pregnancy Prevention Programme here, I'll send it to you in the post…”, I never received it…I don't know when I last signed one. (Lydia)

### Theme 3: Motivation

Finally, three subthemes of motivation were identified.

#### Subtheme 1 Reflective (Barrier): Lack of patient empowerment and self‐efficacy (Reflective Barrier)

Participants often reported a perceived power imbalance between themselves and their valproate‐prescribing HCP. Many felt they were not encouraged or supported to engage fully in discussions about their antiseizure medication, reproductive health, or contraceptive choices.

You think they know better than yourself…if you go in by yourself, you're sometimes a bit afraid to speak up because you think a doctor knows better. (Kate)

One participant shared her frustration about the difficulty in finding a doctor willing to perform permanent sterilization, despite her absolute and resolute decision not to have more children:

It took 18 months to find a doctor who would sterilise me because [HCPs said] I was ‘too young’. (Tanya)

Participants also felt disempowered when their expressed wishes, such as discontinuing valproate or exploring alternative antiseizure medications, were dismissed. This led to a sense of diminished autonomy over their epilepsy treatment, reproductive goals, and healthcare decisions.

The day that I got my Epilim [valproate] finally changed, I had to take my partner with me. We kind of very much presented this front of like, yes, we're looking at having children and then I was taken seriously. And that must have been like the fourteenth, fifteenth time. I was coming in every time asking [to switch from valproate], just completely dismissed. (Kelly)

#### Subtheme 2 Reflective (Barrier): Negative experiences of inclusion in decision‐making

Participants spoke of experiencing paternalistic healthcare in terms of selective information sharing, assertive behaviours, and HCP inflexibility. A pattern of lack of trust was closely related to HCP paternalistic behaviours and attitudes about treatment plans, notably when HCP inflexibility contradicted patient‐informed reproductive healthcare or antiseizure medication preference and excluded patients from collaborative decision‐making.

Participants noted that experiencing selective information sharing and paternalistic attitudes often contributed to a broader sense of mistrust in their relationships with HCP.

…it made me quite distrustful… I felt upset that they hadn't told me [of the risk of valproate use in pregnancy]… I was quite suspicious… annoyed that they hadn't told me ‘cause I felt it was my right to know. (Bella)

Some participants spoke about paternalistic infringements to their reproductive autonomy, such as the cessation of prescribed valproate against their explicit wishes, in favour of concerns about the speculative event of a [currently inexistent] future child not yet conceived (i.e. hypothetical prenatal entities). Some reflected on the effect of valproate cessation on their quality of life (e.g. seizure control, work, relationships, driving status, independence and mental health).

Sometimes it feels like the life of a hypothetical fetus that I don't plan to have is more important than the health that I'm experiencing now in the real world. (Faye)

Some participants had clearly communicated to their HCPs that they did not intend to become pregnant and that they wished to remain child‐free for life but were not offered the option of discussing long‐acting reversible contraception (LARC) or permanent options that may have aligned with their reproductive goals and could have supported [where clinically appropriate] continued valproate use. Instead, some felt valproate was paternalistically withheld solely because they were of ‘childbearing age’—an experience described as particularly disempowering and distressing by participants who firmly wished to remain permanently child‐free by choice.

He said I had to stop taking it because I was ‘of childbearing age’. But no explanation given to me as to why…I never wanted children…I didn't have the control over my body. (Kate)

The above participant, Kate, transitioned from effective seizure‐controlling duotherapy (valproate and carbamazepine) to a complex polytherapy regimen involving five different antiseizure medications (which excluded valproate), a combination that failed to provide seizure control. Ultimately, Kate underwent temporal lobectomy surgery, and while the surgery was successful, Kate was left questioning whether years of uncontrolled seizures and the need for invasive surgery could have been avoided had her original valproate (and carbamazepine) prescription not been refused. Given her consistent, immutable, and unequivocal desire to remain child‐free for life, Kate reflected on the emotional and clinical consequences she experienced when her seizure control appeared secondary to safeguarding against a theoretical pregnancy. She described this as a deeply distressing burden—one shaped by systemic caution and clinical protocols that, in her view, did not fully consider her individual preferences, values or reproductive goals.

#### Subtheme 3 Automatic (Facilitator): Emotional impact of past adverse reproductive health outcomes and future decision‐making

Some participants had previously become pregnant while taking valproate and shared their experiences of having a child or children diagnosed with foetal valproate spectrum disorder (FVSD), while others described experiencing pregnancy loss, including miscarriage and stillbirth, during valproate treatment. Some participants shared that their children diagnosed with FVSD faced a range of challenges, including physical disabilities, neurodevelopmental disorders and/or behavioural difficulties, with varying presentations. For many, their family planning goals were irrevocably altered by the birth of a child with FVSD or the loss of a pregnancy. None of the participants with a child diagnosed with FVSD wished to have more children.

I'm not going to have any more children. I've accepted that…I don't want another future baby to be another statistic. (Lydia)

One participant shared the devastating experience of losing her only child, with FVSD recorded as a contributing factor in his death. She conveyed the intense, enduring emotional pain, the shattering of her world, and the profound grief that followed. The deeply traumatic experience sparked an overwhelming desire to raise awareness of FVSD and the risks of valproate use during pregnancy. She also shared how her reproductive goals had been permanently changed following the birth of her son.

If my son hadn't been born with valproate syndrome, I would have had more children…it wasn't something that I wanted to go through again, and I couldn't have put another child through it… I just didn't want to put another child through what [my son] would have been going through. (Tanya)

Following the stillbirth of her first child who was exposed to valproate in‐utero, another participant recalled feelings of hopelessness and overwhelming loss when considering her reproductive health care goals, and fragile, precarious plans and aspirations for the future.

I seriously considered, you know, if I have to stay on this medication, [if] there's no other options, then children are just not going to be an option. (Isobel)

Some participants spoke about wishing others (e.g. other patients, patient groups, HCP or epilepsy support organizations) had shared accounts (story sharing) of FVSD and the importance of preconception planning with them while they were in the pre‐pregnancy‐contemplation stage of their reproductive health care journeys.

There's probably going to be certain ages, where they'll be thinking, I don't care about that, I'm not having a baby anytime soon. And actually, they need to realise that they need to be doing that now for their 10‐year older self. (Chloe)

## DISCUSSION

This study utilized the COM‐B theoretical framework to identify barriers and facilitators to SDM in valproate prescribing from the perspectives of women with epilepsy and pregnancy potential aged 18–50 years. While participants strongly welcomed SDM as a means to align antiseizure medication use with individual reproductive health care goals, they often reported poor experiences with reproductive health care information exchanges with HCPs, inadequate information about valproate risks, and poor support for reproductive health and goal planning. Through the COM‐B framework lens, these issues challenge the domain of capability to engage in SDM. Additionally, participants struggled with navigating competing priorities, such as seizure control versus reproductive health, had limited information about LARC options, and often lacked the skills needed to discuss highly effective contraception and reproductive goal planning. These factors further impacted the capability domain in relation to engaging in SDM.

A lack of opportunity for preference‐based collaborative discussion impacted the COM‐B domain of opportunity, as did limited access to reproductive health services (e.g. epilepsy preconception services) and limited physical resources (such as consultation duration). Negative (reflective) barriers to motivation included the perceived lack of empowerment and negative experiences of inclusion in treatment planning. Participants frequently reported lacking self‐efficacy and less collaborative involvement with their HCP for treatment decisions than they would wish (reflective barriers to SDM engagement). Consistent with previous research (Pourgholam et al., 2022), motivation to engage in SDM was undermined by perceived disempowerment, low self‐efficacy and paternalistic clinical encounters.

Past experiences, or knowledge of, adverse reproductive health outcomes, including those involving personal experience of FVSD, were described as deeply, deeply distressing, and for some participants, these experiences acted as a powerful motivational force to demand more collaborative and transparent approaches to decision‐making. Such emotionally charged reflections may represent a form of automatic motivation, where deeply felt emotional responses to previous events have led to a heightened desire (from negative associative learning) for SDM in future treatment planning (Jackson et al., 2014; Michie et al., 2011). Additionally, reflective motivation of those not personally impacted by FVSD may be influenced by beliefs about future adverse events and outcome expectancies, motivating participants to demand more collaborative decision‐making approaches (Jackson et al., 2014; Michie et al., 2011). Participants welcomed the premise of comprehensive, collaborative SDM discussions with HCPs both at valproate initiation and throughout treatment, as also advocated by the Independent Medicines and Medical Devices Safety Review [Cumberlege Report] (2020), the Patient Safety Commissioner (Hughes, 2023), the Epilepsy Society (2023) and Harris et al. (2020).

Findings from the present study echo those from systematic qualitative evidence synthesis of routine LARC provision in general primary care (Linton et al., 2023). This study's findings also align with previous research on reproductive healthcare advice given to women with epilepsy who later became pregnant while prescribed valproate (Beardsley et al., 2021; Gosset et al., 2022). A comprehensive effort is needed to provide more opportunities for SDM for women with pregnancy potential when considering valproate as a treatment option.

While patient‐facing educational resources are important, they do not replace high‐quality, collaborative conversations with HCPs that provide an opportunity to deliver individualized epilepsy and reproductive health care planning; effective SDM demands more than information alone (Harris et al., 2020; Rock, 2021; Widnes et al., 2012).

### Implications for policy and practice

This study highlights the importance of timely and ongoing opportunities for SDM when initiating or reviewing valproate prescribing to women with pregnancy potential, especially as reproductive goals evolve. Given the complex balance between seizure management and reproductive health, SDM should be incorporated as a standard component of epilepsy care (NICE, 2021b; NICE, 2022). The timing of reproductive healthcare planning is also critical to meeting the aims of preconception counselling—optimizing maternal health before conception, reducing in‐utero risks, and enabling informed, collaborative reproductive decisions for women with epilepsy (Winterbottom, 2012).

SDM is a key objective across multiple UK health policies and is increasingly seen as the gold standard in care delivery where more than one treatment option is clinically appropriate (Department of Health and Social Care, 2021a; NHS, 2019; NICE, 2019, 2021a). As such, a robust policy framework exists to support the adoption of SDM in UK epilepsy care pathways (NICE, 2023). SDM is not consistently implemented in practice (Department of Health and Social Care, 2021a), but existing touchpoints, such as epilepsy clinics, annual reviews, general primary care and sexual health services, may afford opportunities to embed SDM within epilepsy care pathways. However, there are significant financial and resource barriers to the implementation of SDM in overstretched health systems (Hughes, 2023). These include an ageing population with higher comorbidities, long waiting lists and the significant resources already expended to implement additional valproate‐related regulations and risk minimization measures (MHRA, 2023a, 2023b; Pel‐Littel et al., 2021; Waddell et al., 2021).

Evidence‐based patient decision aids could further facilitate SDM by providing knowledge and opportunities to consider and balance the risks and benefits of valproate within the context of an individual's reproductive goals (Department of Health and Social Care, 2021b; Leech et al., 2021; NICE, 2021a/2021b), particularly when designed with patient input (Joseph‐Williams et al., 2014). Support tools that facilitate SDM with HCPs exist for other chronic conditions involving reproductive health considerations. For example, ‘My Voice CF’ is a web‐based, preference‐sensitive decision aid developed to enhance reproductive planning and patient engagement among women with cystic fibrosis (Stransky et al., 2022).

Epilepsy/valproate‐specific decision aids, developed collaboratively with patients and rigorously evaluated, could support tailored, person‐centred and preference‐sensitive care. Such SDM tools can also provide clinicians with structured guidance when counselling women about treatment options, helping to ensure that discussions are balanced, consistent and aligned with individual reproductive and seizure control goals. In this way, SDM tools have the potential to empower patients and their HCPs to make fully informed, person‐centred treatment decisions; however, adequate financial and staffing resources are needed to allow sufficient clinical time for these discussions (Hanna et al., 2024).

Additionally, training in highly effective contraception could help epilepsy teams better advise patients on optimal contraception methods, particularly LARC. Study findings indicate that participants experienced limited access to such support, and improving training could further support longer‐term, more effective pregnancy planning. Furthermore, participants' experiences in this study highlighted the need for more equitable psychological support for those no longer able to pursue reproductive goals and for those impacted by the birth or loss of a child affected by FVSD.

### Strengths and limitations

This study addresses the gap in the literature regarding women's experiences of SDM at the intersection of epilepsy, valproate prescription and reproductive health. To the authors' knowledge, this is the first COM‐B theory‐informed qualitative study exploring the capability, opportunity and motivational barriers and facilitators to accessing SDM for women with pregnancy potential prescribed valproate. Participants were geographically dispersed across the UK, with representation from multiple constituent nations, helping mitigate single‐centre bias or localized findings and enhancing the robustness and broader relevance of the findings. Participants represented diversity in age, family planning status and reproductive goals, and while the sample of participants was small, the study was guided by the principle of information power rather than traditional saturation (Malterud et al., 2016), supporting the depth and relevance of the insights gathered.

Limitations of this study include that the data were coded by a single researcher, which may increase the potential for subjective interpretation and reduce the trustworthiness and rigour of qualitative research. However, the rigour of the analysis was enhanced through reflexive, iterative engagement and collaborative discussions with the multidisciplinary research team, which served as a triangulation method to refine and evaluate themes (Barbour, 2001; Clarke & Braun, 2013; Liamputtong & Ezzy, 2005; Robson, 2002). Further limitations to the study include a lack of representation of women with epilepsy from ethnic and gender minorities. It has been demonstrated that individuals from minority groups may experience culturally specific societal stigma and cultural differences in perception and experiences around epilepsy resulting in specific social restrictions (Mayor et al., 2022). Further, the focus of this study was on adult women with capacity for consent, but these issues also affect children (and their parents/carers), adolescents and adults with additional support needs; future research is needed to understand their experiences. It is important to recognize that individuals with additional support needs may face further challenges in participating in SDM, which highlights the need for accessible, supportive communication to facilitate equitable, person‐centred care (Davies et al., 2020; Tuffrey‐Wijne et al., 2013; Watkins, Cock, Angus‐Leppan, Morley, et al., 2019; Watkins, Cock, Angus‐Leppan, & Shankar, 2019). The authors highlight the need for further research to explore the findings' broader applicability and to identify specific issues that may impact individuals across diverse social and cultural contexts; future research is needed to understand these patient experiences.

Moreover, there is a potential for recruitment bias towards women with negative experiences of valproate treatment or personal experience of FVSD. It is also important to consider that some participants may have initially been prescribed valproate before the MHRA's progressively strengthened safety communications and prescribing restrictions, which have become increasingly stringent over time (MHRA, 2024).

## CONCLUSIONS

Decision‐making around valproate is inherently complex and requires thoughtful, individualized discussions that consider both the risks of withholding and benefits of prescribing for seizure control in the context of each patient's individual circumstances, preferences, and reproductive goals (Angus‐Leppan & Liu, 2018). However, SDM takes time and expertise; patient decision aids can support both patients and healthcare providers to engage in SDM (Elwyn et al., 2012; Joseph‐Williams et al., 2024).

This research offers a novel exploration through the COM‐B lens of the potentially modifiable capability, opportunity and motivational factors influencing participation in SDM for women with pregnancy potential prescribed valproate. The diversity and complexity of participant reproductive healthcare goals, values and preferences highlight the need for individualized, equitable, responsive SDM whenever valproate is a clinically appropriate option. Fostering a culture that supports SDM, offering regular, timely and ongoing opportunities for engagement, providing epilepsy HCPs with specialized reproductive healthcare training, and providing patient decision aids may all have utility for enhancing SDM participation in epilepsy care. Further research with clinical and patient expert input is needed to co‐design and evaluate SDM interventions to improve the quality of care for women with pregnancy potential when considering valproate treatment for epilepsy.

## NOTE ON GENDER INCLUSIVITY

For the purpose of this paper (for clarity and ease of reading), the term ‘women’ has been used to describe people aged over 18 years who were assigned female at birth. The term ‘women’ will help avoid ambiguity in the communication of the research findings (particularly as the Journal of British Health Psychology has international reach and the term is universally recognized and understood across different cultures). However, the present study was open to all people assigned female at birth who met the stipulated study inclusion requirements.

## AUTHOR CONTRIBUTIONS

**Sarah Louise Griffiths:** Investigation; writing – original draft; methodology; writing – review and editing; project administration; conceptualization; resources; validation; visualization; formal analysis; data curation. **Delyth James:** Conceptualization; funding acquisition; writing – review and editing; project administration; supervision; resources; methodology; validation; formal analysis; data curation; visualization. **Denitza Williams:** Writing – review and editing; visualization; formal analysis. **Lynette James:** Conceptualization; funding acquisition; writing – review and editing; visualization. **Andrew Evans:** Conceptualization; funding acquisition; writing – review and editing; visualization. **William O. Pickrell:** Writing – review and editing; visualization. **Christine McKnight:** Writing – review and editing; visualization. **Sarah Brown:** Writing – review and editing; visualization. **Rhiannon Phillips:** Conceptualization; funding acquisition; writing – review and editing; project administration; supervision; resources; methodology; validation; formal analysis; data curation; visualization.

## FUNDING INFORMATION

This study was supported by a project grant from the Welsh Government.

## CONFLICT OF INTEREST STATEMENT

Andrew Evans declares that he is a member of the Commission on Human Medicines Valproate Implementation Advisory Expert Working Group. William O. Pickrell declares that he has received an unrestricted research grant and speaker fees from UCB Pharma, as well as speaker fees from Angelini Pharma.

## ETHICS STATEMENT

The Cardiff School of Sport and Health Sciences granted ethical approval for the study under the Cardiff Metropolitan University Ethics Framework 27/09/2022. Project reference number: PGT‐6089.

## PATIENT AND PUBLIC INVOLVEMENT (PPI)

Two PPI contributors provided critical input into the revision of this manuscript. They were not involved in the study design, data analysis, interpretation of findings or writing of the manuscript but contributed valuable feedback to support the accessibility of the final manuscript. The authors gratefully acknowledge and thank them for their invaluable input.

## DISCLOSURE STATEMENT

This manuscript is based in part on a Health Psychology Master's dissertation by Sarah L Griffiths, titled *Developing a Shared Decision‐Making Tool for Sodium Valproate Prescribing for Individuals of Childbearing Potential*, submitted to Cardiff Metropolitan University in May 2023. The dissertation is unpublished but available from the Cardiff Metropolitan University institutional repository upon request. The present manuscript includes findings from a larger participant group and expands upon the original research. Findings from this study were presented to the Welsh Government and subsequently informed a two‐phase, multidisciplinary valproate‐specific research and stakeholder engagement project. This subsequent work led to the development of a patient decision aid, now available on the NHS All Wales Therapeutics and Toxicology Centre website and is freely accessible for use in NHS clinical settings across the UK (All Wales Therapeutics and Toxicology Centre, 2024). The patient decision aid can be accessed at https://awttc.nhs.wales/files/guidelines‐and‐pils/patient‐decision‐support‐leaflet‐making‐a‐decision‐about‐valproate‐eng‐pdf/.

## Supporting information


File S1.

## Data Availability

Data are not publicly available due to privacy and ethical restrictions. However, anonymised data may be made available from the corresponding author upon reasonable request and subject to review by the research team or relevant ethics committee to ensure compliance with ethical standards.
