# Supplementary material for: Capability, opportunity and motivation for shared decision‐making about valproate as an antiseizure medication treatment for epilepsy in women with pregnancy potential: A qualitative study of patient perspectives
Source: Br J Health Psychol. 2025 Dec 19;31(1):e70045. doi: 10.1111/bjhp.70045 (PMC12717438; doi:10.1111/bjhp.70045)
Supplement: Supplementary file 1 — File S1. [file BJHP-31-0-s001.docx]

**Supplementary File 1: Semi-structured Interview Schedule**

**Interview Schedule**

**Developing a shared decision-making tool for sodium valproate prescribing for individuals of childbearing potential**

General prompts for use at any point during the interview

*Can you tell me more about that?*

*How does that make you feel?*

*Can you give an example?*

*Why would you say that is?*

*That’s interesting; what makes you say that?*

*Is there anything else you would like to suggest?*

*Are there any reasons why you think this might / might not help?*

Reminder:

1. Seek out what is most important to the participant.
2. Be led by the participant.
3. Focus on what is important to them.

Adopt a narrative approach and encourage the participant to tell their story.

Interview Introduction

1. Please start by introducing yourself.
2. Current role and purpose of interview.
3. Purpose of the study

Part 1: Timeline

Introduce the timeline. Has the participant had a chance to complete the timeline, or would they like to look at the timeline now?

Ask the participant if they are happy for you to have a copy of their timeline (emailed or screenshot) to help you understand their story. (Alternatively, post at the end of the interview).

Use prompts (above) where appropriate throughout the interview and invite the participant to add to the timeline whenever they wish. Explore each relevant question point but be fluid and led by the participant.

1) Can you tell me a little about your experience of epilepsy?

- When did epilepsy start? Age/stage of life. Timeline

Now I’d like to talk to you about your epilepsy medication sodium valproate (you may know it as Dyzantil, Epilim, Episenta, Epival) (Find out which name participant is familiar with and use throughout interview).

2) How do you/did you feel about using valproate?

- How long has the participant been prescribed valproate? Age/stage of life when first prescribed?
- Any concerns about using valproate?
- Has it affected reproductive health goals/preferences? (e.g., family planning, contraception choice) (check with timeline if provided).

3) How well informed do you feel you have been about using sodium valproate and the risks of its use in pregnancy? Do you feel that you have had:

- - too much information
  - enough information.
  - not enough information.
  - I don’t need any information; I rely on my healthcare provider to tell me what medicines I need to take to control my epilepsy.

Part 2: Information Video Regarding Valproate

Next, I’d like to show you a short information video produced by the Welsh Government. The government in Wales wants to make sure that the best support is given to people with epilepsy. In particular when the medicine valproate is used for individuals who could become pregnant. I’ll show you the video and then ask you a few questions about it.

Show video

4) What do you think about the video?

- Anything particularly liked/disliked?

5) When do you think this video would be most useful to show individuals with childbearing potential who are prescribed/talking about valproate?

- (e.g., first specialist consultation, annual review, pharmacy prescription service as an ongoing reminder).
- Where/how: In clinic? At review? Pharmacy dispensing-repeat prescription (e.g., QR code on box)?
- Thoughts on visual delivery of vital information. Acceptability. Usability.
- Thoughts on easily accessed/digestible information. E.g., video.

6) Where do you think this video would have, or could now, fit into your valproate care pathway or experience? Explore.

7) Is there any information from the video you wish you knew earlier/sooner? Timeline

8) After watching the video, how well informed do you now feel you were previously about using valproate and the risks of use in pregnancy?

Things to follow up on if relevant (if participant has children/indicated a desire to have children). Be sensitive.

- Family/children: has valproate use influenced reproductive health choices?
- What (if any) preconception guidance/counselling regarding valproate use did/would they seek before pregnancy contemplation/planning stages? Have they heard about preconception counselling? Was this offered/available to them?

9) In addition to the information in the video, what else does the participant think can be done to support individuals with childbearing potential to decide about taking valproate?

- What else may the participant have benefitted from? (Explore the timeline).

10) From their experience, how is information about valproate use and the pregnancy prevention programme shared with patients?

- What do conversations with healthcare providers really look like? What has their experience looked like.
- What are their experiences of reproductive choice support (Pregnancy Prevention Programme/contraception options/access to counselling)?
- Who is their preferred healthcare provider in relation to reproductive health choice questions/information finding (epilepsy/neurologist specialist, epilepsy nurse, GP etc.)

Part 3: Experiences with Epilepsy Specialist

11) How would the participant describe their relationship with their valproate prescribing epilepsy specialist?

Prompt: Do they feel comfortable/able to contact their specialist or preferred healthcare provider if they had any concerns/questions about valproate and reproductive health choices? (Check timeline and stages of life).

- Role of epilepsy specialist/nurse/ pharmacist. Experiences of the valproate delivery stages. Most accessible/approachable?

Part 4: Support in Decision-Making (Shared Decision-Making Experiences)

12) How well do they feel their healthcare provider involved them in deciding if valproate was the right treatment for them considering their reproductive health choices, goals and preferences?

13) How well does participant feel their specialist listened/listens if they have questions or concerns about valproate use and their reproductive health?

14) Has the participant heard about shared decision making? (If not provide a brief explanation.)

15) Did friends/ family/significant other help with decisions about if valproate was the right option (when/if valproate was recommended by HCP)?

16) How well supported by their epilepsy healthcare provider did the participant feel in making the decision to start taking valproate?

- How much were individual’s reproductive health choices or preferences considered by the specialist when patient/HCP were planning treatment?
- How involved was the participant in deciding on antiseizure medication? Was this by choice? (E.g., did the participant choose to rely on HCP or did they want to be actively involved in the treatment planning).

17) Can participant think of anything that could have helped support them more meaningfully (or could help support another woman) to decide about whether to start taking valproate?

- What does support/input from healthcare provider (consultant)/epilepsy nurse/GP/pharmacist) look like (if support was provided)?

18) Is there anything that has not been covered that the participant would like to talk about, or is there anything they would like to add?

19) Would participant be happy for the interviewer (or a member of the research team) to contact them in the future to look at decision aid support tools that may help patients decide if valproate is the right epilepsy medication for them when considering their reproductive health choices?

Part 5: Closing

Thank the participant for taking part in this study.

Is there anything the participant is concerned or worried about and would like to talk about before finishing the interview?

If the participant has any concerns, please address them. Remind the participant that there are some helplines on the participant information sheet. Moreover, if the participant wants to discuss anything about the interview over the coming days, they are more than welcome to contact interviewer or the project supervisors who would be more than happy to speak to them about any queries.

Please ask the participant to contact their GP or specialist healthcare provider if they feel they need advice regarding their epilepsy care or antiseizure medication management.

Remind participant that it is really important not to stop or change anything about their prescription medication without first speaking to their healthcare provider.

Arrange to get a copy of the completed timeline (if not already in possession of a copy).

Arrange delivery of Amazon voucher

If the participant is satisfied with information, thank them again for their time and the interview can conclude.

**Supplementary File 2: Visual Timeline Templates**

**Figure 1**

*Blank timeline template for participant completion*


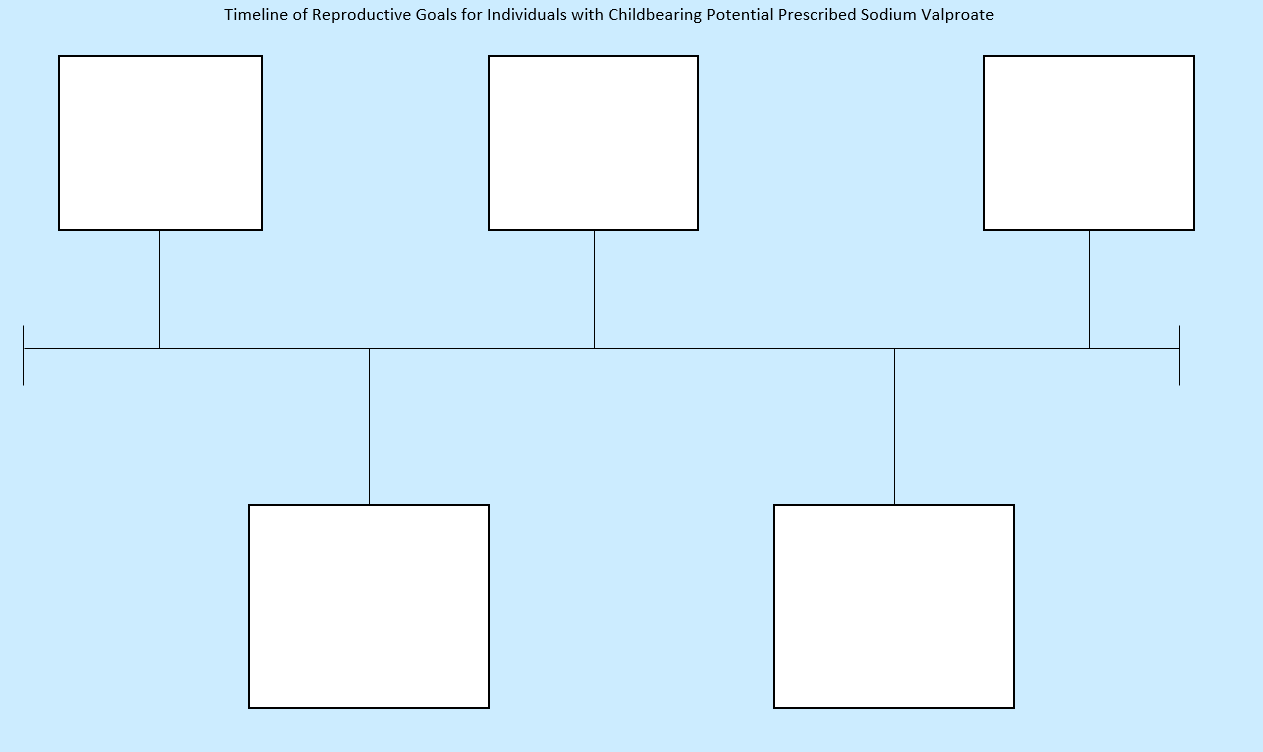


**Figure 2**

*Example completed timeline*


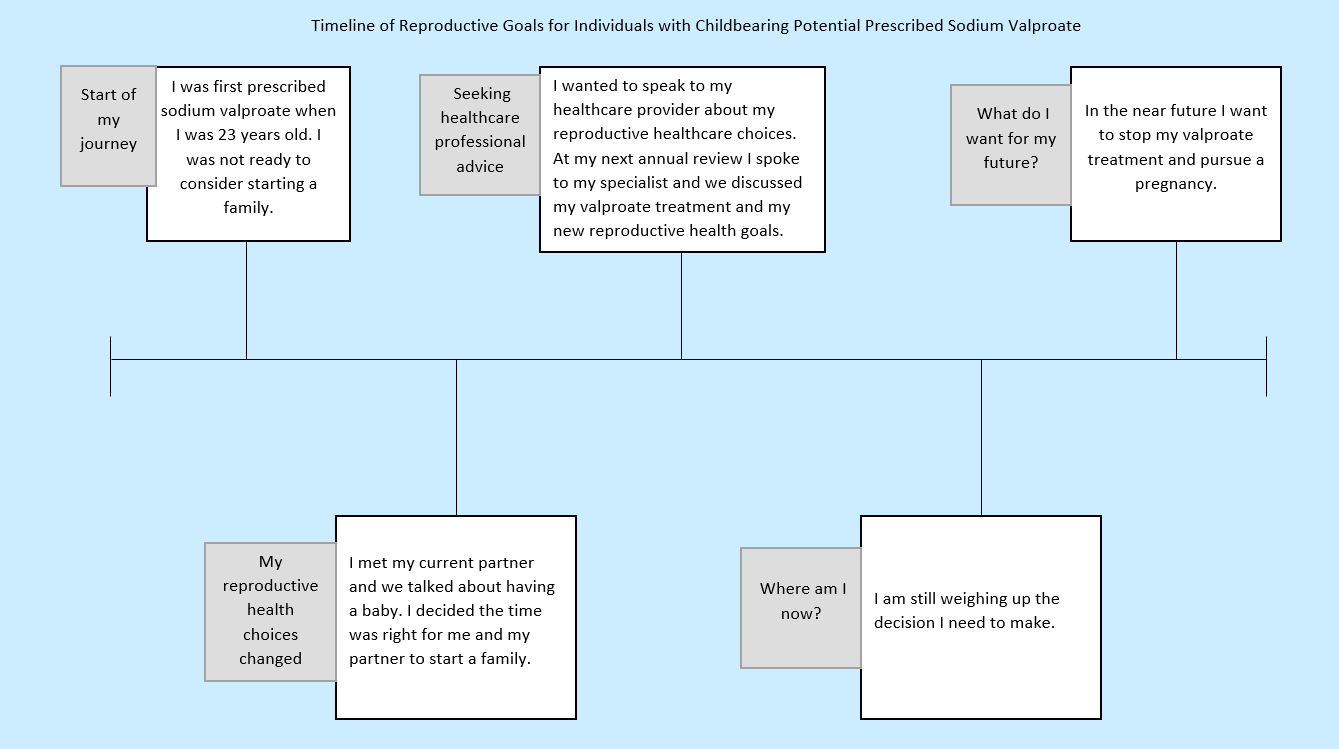


**Supplementary File 3: Reflexivity**

The lead researcher (SG), who conducted all interviews and coded the data, adopted reflexivity throughout the study data collection and analysis to appraise personal involvement and bias (Braun & Clarke, 2022). SG is a White British cisgender woman, was at the time of study in her 40s, and identified with similar life-stage-specific experiences to many of the participants in the study. SG had no personal experience of epilepsy or valproate prescription. However, she could relate to the reproductive life-stage and experiences of many of the participants. Reflecting analytically, she acknowledged that her attitudes, life journey experiences, values and preconceived notions about the participant’s experiences of epilepsy, valproate prescription, seizure control, and experiences of possible clinician/patient power imbalance might influence preconceived perceptions about the narrative expression of the participant's lived experiences.

SG endeavoured to deepen her understanding of the participant’s lived experience to strengthen the robustness of the findings (Macbeth, 2001). SG considered the dynamic of the interviewer/participant relationship and was aware of the need to adopt an unbiased and non-directive approach to the interviews as opposed to a counselling approach. SG had no prior relationship with the interviewees. During interviews, the interviewer/interviewee relationship was open, amenable, warm, and comfortable, facilitating free-flowing dialogue. Co-authors had no direct research contact with interviewees but contributed to the development of the coding framework and interpretation of findings.

Two of the authors are cisgender males, and the remaining authors are cisgender females. They are from differing professional backgrounds, including health psychology, sexual health outreach, pharmacy, patient safety, health policy, and (clinically practicing) neurology.

**Supplementary File 4: COREQ Checklist**

**Consolidated criteria for reporting qualitative studies (COREQ): 32-item checklist**

| No | Item | Guide question/description | Page number |
| --- | --- | --- | --- |
| **Domain 1: Research team and reflexivity** | | | |
| Personal  Characteristics | | | |
| 1. | Interviewer/facilitator | Which author/s conducted the interview or focus group? | 11 |
| 2. | Credentials | What were the researcher's credentials? E.g. PhD, MD | 11 |
| 3. | Occupation | What was their occupation at the time of the study? | 11 |
| 4. | Gender | Was the researcher male or female? | 11 |
| 5. | Experience and training | What experience or training did the researcher have? | 11 |
| Relationship  with  participants | | | |
| 6. | Relationship established | Was a relationship established prior to study commencement? | 11 |
| 7. | Participant knowledge of the interviewer | What did the participants know about the researcher? e.g. personal goals, reasons for doing the research | 11 |
| 8. | Interviewer characteristics | What characteristics were reported about the interviewer/facilitator? e.g. Bias, assumptions, reasons and interests in the research topic | 50 (Supple-mentary File 3) |
| **Domain 2: study design** | | | |
| Theoretical  framework | | | |
| 9. | Methodological orientation and Theory | What methodological orientation was stated to underpin the study? e.g. grounded theory, discourse analysis, ethnography, phenomenology, content analysis | 8, 10, 12, 13 |
| Participant  selection | | | |
| 10. | Sampling | How were participants selected? e.g. purposive, convenience, consecutive, snowball | 9 |
| 11. | Method of approach | How were participants approached? e.g. face-to-face, telephone, mail, email | 9, 10 |
| 12. | Sample size | How many participants were in the study? | 14 |
| 13. | Non-participation | How many people refused to participate or dropped out? Reasons? | 14 |
| Setting |  |  |  |
| 14. | Setting of data collection | Where was the data collected? e.g. home, clinic, workplace | 11 |
| 15. | Presence of non-participants | Was anyone else present besides the participants and researchers? | 11 |
| 16. | Description of sample | What are the important characteristics of the sample? e.g. demographic data, date | 14 (Figure 1) |
| Data  collection | | | |
| 17. | Interview guide | Were questions, prompts, guides provided by the authors? Was it pilot tested? | 10 |
| 18. | Repeat interviews | Were repeat interviews carried out? If yes, how many? | 12 |
| 19. | Audio/visual recording | Did the research use audio or visual recording to collect the data? | 12 |
| 20. | Field notes | Were field notes made during and/or after the interview or focus group? | 12 |
| 21. | Duration | What was the duration of the interviews or focus group? | 14 |
| 22. | Data saturation | Was data saturation discussed? | 10, 30 |
| 23. | Transcripts returned | Were transcripts returned to participants for comment and/or correction? | 12 |
| **Domain 3:**  **analysis and**  **findings** | | | |
| Data analysis |  |  |  |
| 24. | Number of data coders | How many data coders coded the data? | 12 |
| 25. | Description of the coding tree | Did authors provide a description of the coding tree? | 13 |
| 26. | Derivation of themes | Were themes identified in advance or derived from the data? | 12, 13 |
| 27. | Software | What software, if applicable, was used to manage the data? | 12 |
| 28. | Participant checking | Did participants provide feedback on the findings? | N/A |
| Reporting | | | |
| 29. | Quotations presented | Were participant quotations presented to illustrate the themes / findings? Was each quotation identified? e.g. participant number | Yes (16–27) |
| 30. | Data and findings consistent | Was there consistency between the data presented and the findings? | Yes (16-27) |
| 31. | Clarity of major themes | Were major themes clearly presented in the findings? | Yes (16-31) |
| 32. | Clarity of minor themes | Is there a description of diverse cases or discussion of minor themes? | Yes (17-31) |

**Supplementary File 5**

**The Contributor Role Taxonomy (CRediT)**

| Term | Definition | Name |
| --- | --- | --- |
| Conceptualization | Ideas; formulation or evolution of overarching research goals and aims | Andrew Evans, Lynette James, Delyth James, Rhiannon Phillips, Sarah Griffiths. |
| Methodology | Development or design of methodology; creation of models | Sarah Griffiths, Delyth James, Rhiannon Phillips. |
| Software | Programming, software development; designing computer programs; implementation of the computer code and supporting algorithms; testing of existing code components | N/A |
| Validation | Verification, whether as a part of the activity or separate, of the overall replication/ reproducibility of results/experiments and other research outputs | Sarah Griffiths, Delyth James, Rhiannon Phillips. |
| Formal analysis | Application of statistical, mathematical, computational, or other formal techniques to analyze or synthesize study data | Sarah Griffiths, Delyth James, Rhiannon Phillips, Denitza Williams. |
| Investigation | Conducting a research and investigation process, specifically performing the experiments, or data/evidence collection | Sarah Griffiths. |
| Resources | Provision of study materials, reagents, materials, patients, laboratory samples, animals, instrumentation, computing resources, or other analysis tools | Sarah Griffiths, Delyth James, Rhiannon Phillips. |
| Data Curation | Management activities to annotate (produce metadata), scrub data and maintain research data (including software code, where it is necessary for interpreting the data itself) for initial use and later reuse | Sarah Griffiths, Delyth James, Rhiannon Phillips. |
| Writing - Original Draft | Preparation, creation and/or presentation of the published work, specifically writing the initial draft (including substantive translation) | Sarah Griffiths. |
| Writing - Review & Editing | Preparation, creation and/or presentation of the published work by those from the original research group, specifically critical review, commentary or revision – including pre-or postpublication stages | Sarah Griffiths, Delyth James, Rhiannon Phillips, Denitza Williams, Lynette James, Andrew Evans, William O Pickrell, Christine McKnight, Sarah Brown. |
| Visualization | Preparation, creation and/or presentation of the published work, specifically visualization/ data presentation | Sarah Griffiths, Delyth James, Rhiannon Phillips, Denitza Williams, Lynette James, Andrew Evans, William O Pickrell, Christine McKnight, Sarah Brown. |
| Supervision | Oversight and leadership responsibility for the research activity planning and execution, including mentorship external to the core team | Delyth James, Rhiannon Phillips. |
| Project administration | Management and coordination responsibility for the research activity planning and execution | Sarah Griffiths, Delyth James, Rhiannon Phillips. |
| Funding acquisition | Acquisition of the financial support for the project leading to this publication | Andrew Evans, Lynette James, Delyth James, Rhiannon Phillips. |
